# An Adaptive Data Collection Algorithm Based on a Bayesian Compressed Sensing Framework

**DOI:** 10.3390/s140508330

**Published:** 2014-05-09

**Authors:** Zhi Liu, Mengmeng Zhang, Jian Cui

**Affiliations:** College of Information Engineering, North China University of Technology, Beijing 100144, China; E-Mails: zmm@ncut.edu.cn (M.Z.); landrew@126.com (J.C.)

**Keywords:** Bayesian compressed sensing, routing structure, wireless sensor networks

## Abstract

For Wireless Sensor Networks, energy efficiency is always a key consideration in system design. Compressed sensing is a new theory which has promising prospects in WSNs. However, how to construct a sparse projection matrix is a problem. In this paper, based on a Bayesian compressed sensing framework, a new adaptive algorithm which can integrate routing and data collection is proposed. By introducing new target node selection metrics, embedding the routing structure and maximizing the differential entropy for each collection round, an adaptive projection vector is constructed. Simulations show that compared to reference algorithms, the proposed algorithm can decrease computation complexity and improve energy efficiency.

## 1. Introduction

For data collection Wireless Sensor Networks (WSN), since sensor nodes are always densely distributed, there exists abundant redundancy in data from neighboring nodes. During the data collection process, besides how to route data to the sink, one of the key problems is how to remove redundancy and improve energy efficiency. There are a large number of algorithms that have integrated data compression into data collection [1–6]. Work described in [1,2] uses aggregation functions (e.g., *Max*(·), *Min* (·), *Average*(·), *etc.*) to extract the required information during data routing. However, while data size is reduced, data structure is lost. In [3,4] the authors adopt lifting schemes to compress data during routing. These schemes need to exchange intermediate coefficients between nodes during compression, which results in a waste of energy. Many traditional data compression algorithms such as KLT [5], wavelet [6], data mining [7], have been used in data collection. These algorithms have high computational complexity, and are difficult to integrate with routing processes. Compressed Sensing (CS) [8] is a new emerging theory in signal processing which has promising prospects to be applied in WSN [9–16]. Firstly, in CS, data sampling and compression can be integrated harmoniously into one step, which meets the requirements of WSN energy efficiency. Secondly, in compressed sensing, the computational complexity of decoding and encoding is highly asymmetrical; encoding complexity is very low, which implies a limitation for sensor nodes with weak computational power and limited energy supplies. Finally, during the encoding process in CS, no original or intermediate data is required to exchange among nodes, which is beneficial for distributed sensor network design.

**Figure 1.**
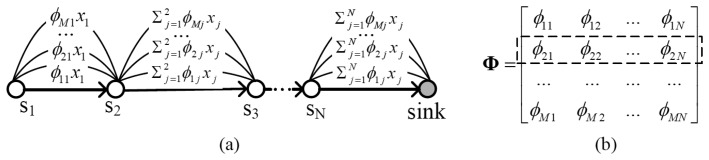
Relationship between routing and projection matrix: (**a**) Routing process; (**b**) Projection matrix.

However, it is not feasible to straightforwardly apply compressed sensing to WSN. Traditional projection matrices are always dense. From Figure 1, one can find that, if the projection matrix is dense, the number of packets transmitted among nodes will be very large. To increase energy efficiency, a sparse projection matrix must be utilized.

There is some work devoted to the design of optimized projection matrices [9–16]. In [12] an iterative algorithm to build a sparse projection matrix and decrease its coherency with the transform matrix was proposed. In each collection round, one randomly chooses one node to begin, and then selects the next node from the previous one's neighbors which can minimize the coherency between these two matrices as the next hop. This procedure loops until a complete route to a sink is formed. Work in [14] defines a metric named mutual-coherency to optimize the projection matrix. Based on this metric, a new iterative scheme is proposed to reduce the coherence step by step. Unlike the aforementioned algorithms, work in [15] suggests the use of Bayesian theory to solve the problem of projection matrix building. The main idea is that, based on some initial observed samples, one may obtain the posterior probability of the underlying signal to aid the construction of the projection vector for the next collection round. The optimization object for the new vector is to maximize the information retrieved in one collection round. However, the projection matrix formulated in this algorithm is always dense, with no routing structure embedded. Based on the framework proposed by [15], work in [16] proposes a greedy algorithm to build a sparse projection vector, which maximizes the information retrieved in one unit of energy consumption. Since the algorithm is greedy, its computation complexity is very high. Furthermore, this algorithm tends to select only one node from a sink's one hop neighbors to build the projection vector, therefore, its reconstruction performance is degraded.

In this paper, on account of these issues, a new adaptive algorithm to design a projection vector to meet the requirements of sparseness and routing structure is proposed. The rest of this paper is organized as follows: a brief description of the Bayesian compressive sensing framework is provided in Section 2. In Section 3, the complete idea of the proposed algorithm is presented. Section 4 gives the simulation results and Section 5 concludes the work.

## 2. Bayesian Compressive Sensing Framework

For an underlying signal vector **x** ∈ **R***^N^*^×1^, which is sparse on basis **Ψ**:

(1) $$\begin{array}{cc} x & =\Psi z\\ \text{s}.\text{t}.{\left\|z\right\|}_{0} & =K,K<<N \end{array}$$

if **x** is noise contaminated, and the observation process does not introduce noise, then the observed data **y** ∈ **R***^M^*^×1^ using projection matrix **Φ** ∈ **R***^M^*^×^*^N^* can be expressed as:

(2) $$y=\Phi (x+e_{s})$$

where *M* ≥ *C*·*K* · log *N*, *M* ≪ *N*, *C* is a constant. **e***_s_* is independent and identically distributed sensing noise.

Based on observed **y**, adopting Bayesian learning theory, the differential entropy of the underlying signal **x** can be obtained as [15,16]:

(3) $$h(x)=-\frac{1}{2}\log|A+\sigma^{-2}\Psi^{T}\Phi^{T}{\left(\Phi \Phi^{T}\right)}^{-1}\Phi \Psi |+\text{const}$$

where *const* is irrelevant to projection matrix, and:

(4) $$\sum ={\left(A+\sigma^{-2}\Psi^{T}\Phi^{T}{\left(\Phi \Phi^{T}\right)}^{-1}\Phi \Psi \right)}^{-1}$$

(5) $$A=\left[\begin{array}{cccc} \alpha_{1} & 0 & \ldots & 0\\ 0 & \alpha_{2} & \ldots & 0\\ 0 & 0 & \ldots & \ldots \\ 0 & 0 & \ldots & \alpha_{N} \end{array}\right]$$

Here *α_i_*, (*i* = 1,… N) is independent and identically distributed hyper-parameter. *σ*^2^ is the noise power. Assuming there are *M*_0_ initial observed samples, to obtain the (*M*_0_ + 1)-th sample, a new projection vector **r***_M_*__0___+ 1_ must be constructed, and the new projection matrix becomes:

(6) $$\Phi_{\text{new}}=\left[\begin{array}{c} \Phi \\ r_{M_{0}+1} \end{array}\right]$$

The information retrieved in the (*M*_0_ + 1)-th collection round can be expressed as [16]:

(7) $$\Delta h(x)=\frac{1}{2}\log(1+\frac{1}{\sigma^{2}}\frac{r_{M_{0}+1}^{T}VV^{T}\Psi \Sigma \Psi^{T}VV^{T}r_{M_{0}+1}}{r_{M_{0}+1}^{T}VV^{T}r_{M_{0}+1}})$$

which indicates the reduction of uncertainty of **x** after a new sample *y_M_*__0___+ 1_ has been received. In Equation (7), **V** ∈ **R***^N^*^×^*^M^*^_0_^ is a matrix acquired from Singular Value Decomposition of **Φ**:

(8) $$\Phi =U\left[\begin{array}{cc} S & 0 \end{array}\right]\left[\begin{array}{c} {\bar{V}}^{T}\\ V^{T} \end{array}\right]$$

It is straightforward that in order to maximize the information retrieved in round *M*_0_ + 1 **r***_M_*__0_ + 1_ should be obtained by solving the following optimization problem:

(9) $$r_{M_{0}+1}=\arg\underset{r}{\max}\frac{r^{T}VV^{T}\Psi \sum \Psi^{T}VV^{T}r}{r^{T}VV^{T}r}$$

It should be noted that, **r***_M_*__0_ + 1_ obtained using the above algorithm is always dense, which means it is not energy efficient. Another problem is that this new derived projection vector does not have a routing structure embedded.

## 3. Adaptive Projection Vector Construction Algorithm

Firstly, we give out some assumptions for the WSN. All sensor nodes are static, and the network is fully connected. The sink knows the neighboring context of each node. As nodes are densely deployed, data from neighboring nodes is highly correlated.

Secondly, we present the constraints an optimal projection vector **r***_M_*__0_ + 1_should meet: (1) The vector should be sparse. (2) Nodes selected in the vector can form a complete routing structure to sink. (3) For all vectors with the same number of nodes selected, the vector constructed by the proposed algorithm can retrieve the maximum information. (4) To meet energy conservation constraint, **r***_M_*__0_ + 1_ should be normalized:

(10) $$\left\{r_{M_{0}+1}\in R^{N},{\left\|r_{M_{0}+1}\right\|}_{2}=1\right\}$$

Since the problem described by (1), (3), (4) is NP-hard [16] and constraint (2) makes it more difficult to solve, we try to find a solution for each constraint and to get a sub-optimal solution for the whole problem. For constraint (1), the key is to select optimal number of nodes—target nodes. Since each non-zero element in the projection vector corresponds to one sensor node, the task here is to find out the nodes from which to harvest information in (*M*_0_ + 1)-th collection round. A proper metric should be defined for node selection. Constraint (2) is supplementary to the first one. When the vector obtained in the first step cannot form a complete routing structure, we need to add the least supplementary nodes to complete it. For condition (3), it is equivalent to determine the optimal projection coefficients for each selected nodes. This can be solved through maximizing the differential entropy of signal **x**.

### 3.1. Determine the Target Nodes

Given a certain number of initial observed samples, in order to determine which nodes need to be harvested the most, we define two metrics from different points of view.

#### 3.1.1. Principle Component Analysis Based Metric

Under the Bayesian compressive sensing framework, one can get the differential entropy of the underlying signal **x**, and then obtain a new projection vector by maximizing the reduction of uncertainty of **x**. However, the vector obtained by this means always has too many non-zero items. Since the direction of a vector is mainly determined by its principle components, we can find out the indices of *L* elements which have the most amplitude in the vector to represent target nodes. For a normalized vector **r** and a given threshold *E_th_*, *L* is determined in the following way:

(11) $$\begin{array}{c} L=\min l\\ s.t.\overset{l}{\underset{k=1}{\text{∑}}}{\left|r_{i_{k}}\right|}^{2}>E_{th},i_{k}\in \{1,\cdots N\} \end{array}$$

#### 3.1.2. Node's Total Coefficients Energy Based Metric

**Figure 2.**
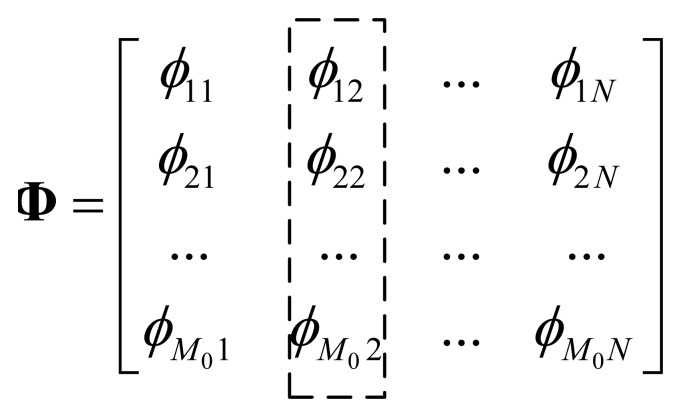
Explanation of node 2's total coefficients energy.

In compressive sensing theory, transform matrix **Ψ** and projection matrix **Φ** of the underlying signal **x** should be incoherent in order to improve reconstruction performance. At the same time, in order to sparse signal **x**, **Ψ** and **x** should have good coherency. That is to say, ideally, **Φ** and **x** should be completely incoherent. Therefore, for all nodes, the statistic feature of their coefficients in **Φ** should be identical, and there is no clue to find out from **Φ** that which node is more or less important. Based on this observation, for each node, we can study its projection coefficients by defining node's total coefficients energy. An example to illustrate the node's total coefficients energy for node 2 is illustrated in Figure 2.

Define (node's total coefficients energy): Given a projection matrix Φ = *φ*_1_, *φ*_2_ ⋯ *φ*_N_, we define the total coefficients energy of node *i* to be the sum of absolute value of *i*-th column elements in matrix Φ:

(12) $$P_{i}=\sum_{j=1}^{M}\left|\phi_{ji}\right|$$

Total coefficients energy *P_i_* indicates the contribution of data from node *i* to the observed samples. Ideally, total coefficients energy for each node should be identical in a statistical sense, however, in an adaptive compressive sensing algorithm, the initial projection matrix has a relatively small number of rows, making the total coefficient energy of some nodes much less than that of others. Therefore, when a new projection vector is to be built, it is reasonable to select these nodes first.

It should be noted that, most projection matrices currently proposed meet the attributes we presented here, *i.e.*, their total coefficients energy for each node is identical in a statistical sense. For example:

(1) Gaussian random matrix: every element in the matrix follows Gaussian distribution with zero mean and 1/*N* variance.
(2) Rademancher matrix: elements of the matrix are selected randomly from set {+1, −1}.
(3) Partial Fourier matrix: randomly select *M* rows from *N*x*N* Fourier matrix.
(4) Cyclic matrix: each row of the matrix is a different arrange of a same set.

### 3.2. Build Routing Structure

#### 3.2.1. Style of the Routing Structure

As stated in the previous subsection, the target nodes selected can't guarantee formulation of a complete routing structure, therefore, some supplementary nodes are needed. These nodes can be used in two styles. One is to use them as routing nodes, and no data is collected from them. The other is to use them both as routing nodes and data collection nodes.

Traditional data collection schemes usually adopt the first style, as adding data collection nodes will increase the transmission packet size, and consume more energy. But in compressive sensing, in one collection round, all data will be projected into a fixed length buffer regardless of the amount of sensors, which means collecting data from routing nodes will not distinctly increase the power consumption. Moreover, there is tangible benefit for this style as it can increase the information collected and decrease the coherency between projection vectors. Therefore, we adopt the second style.

**Figure 3.**
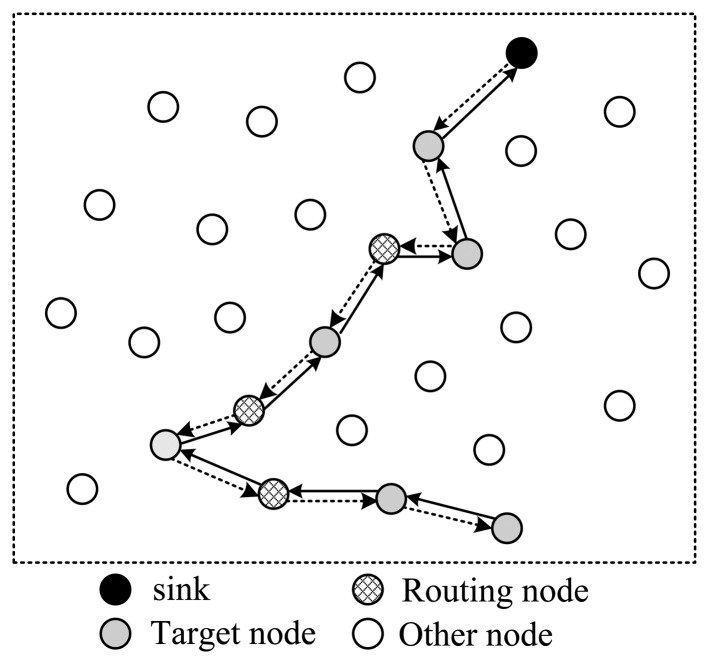
Linear structure.

**Figure 4.**
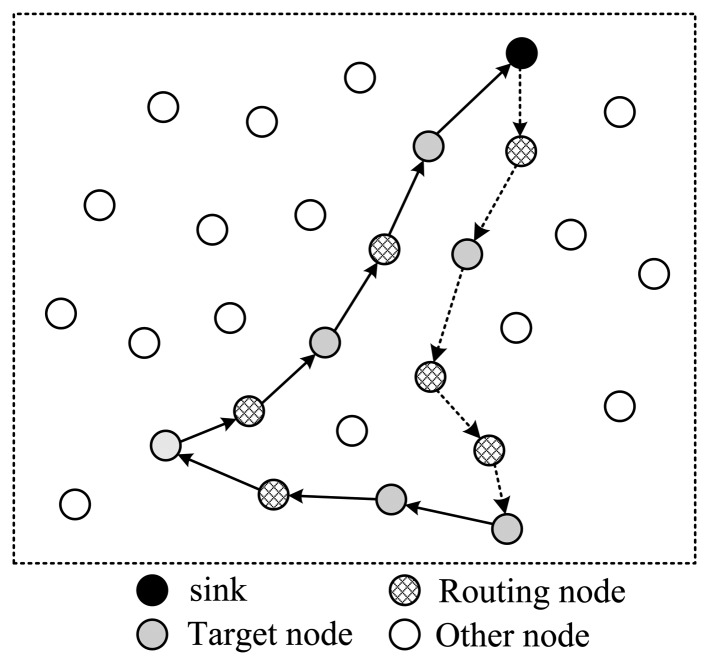
Single-ring structure.

Next, let's discuss how to arrange the selected nodes to form a complete routing structure. In one data collection round, the sink needs to send out packets containing projection coefficients to selected nodes; then the corresponding node will project its data to the projection field of the packet; finally this packet will be routed back to sink. There are several routing structures to complete this procedure, shown in Figures 3 and 4.

In Figure 3, all selected nodes are arranged in a linear structure; the back and forth paths of the packet are the same. In this style, except for the endpoint node, each node will receive and send the packet twice; however, only one time they can project their information into it, which means energy waste. As is shown in Figure 3, there are in total 18 transmissions, but only nine nodes' information is collected.

To improve this, the structure shown in Figure 4 is another option. In this style, all selected nodes are arranged in a ring structure. Every node on the path only needs to transmit the packet once. In the figure, there are totally 13 transmissions, and 12 nodes' information is collected. It's more energy efficient than structure 1. However, this structure leads to a pretty long routing path, making the time needed for one round very long in case of large scale wireless sensor networks.

**Figure 5.**
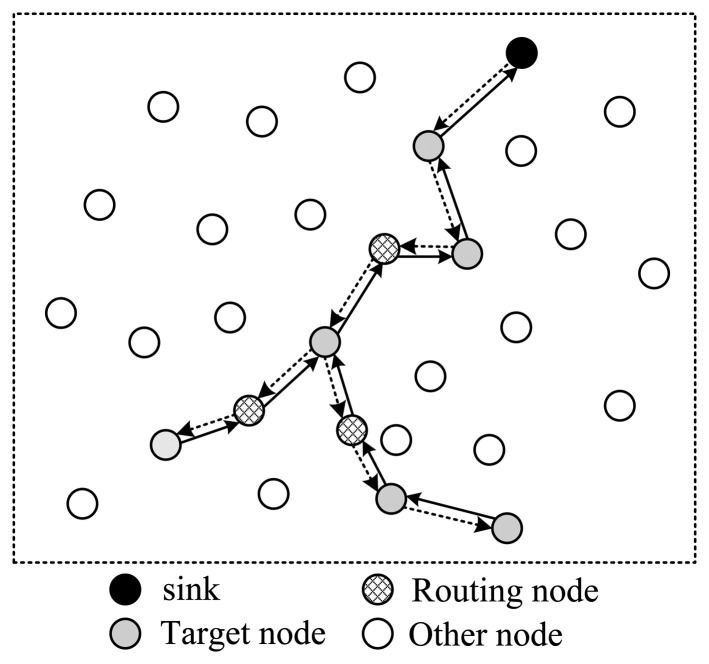
Tree structure.

**Figure 6.**
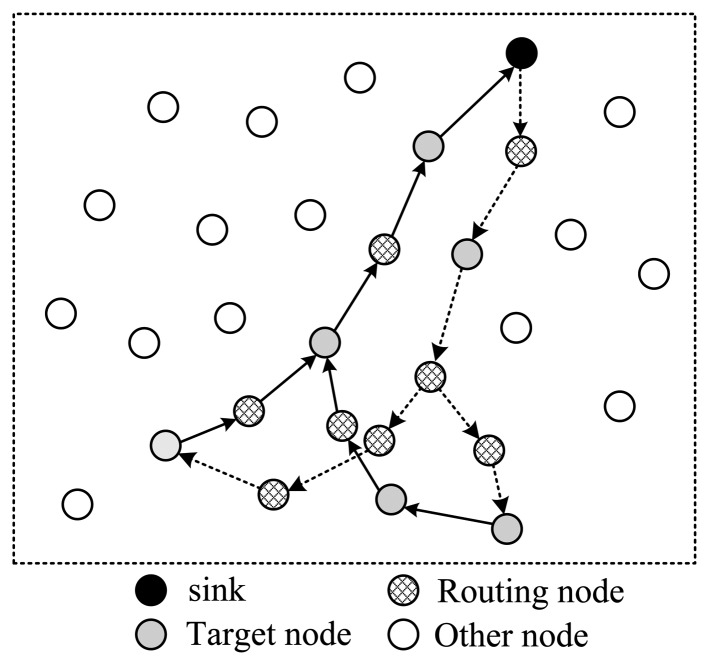
Multi-ring structure.

To shorten the time spent for one collection round, the structure shown in Figure 5 utilizes a tree structure. Like structure 1, data packets pass a same node twice. The structure shown in Figure 6 uses a multi-ring structure to arrange the selected nodes. Packets are duplicated into multiple copies in case of necessity utilizing the broadcast property of wireless channels to speed up the collection process. Meanwhile, multiple copies of the packet are reunited when necessary to save energy. This structure takes the advantage of the structures proposed in Figures 4 and 5, and has the virtue of both energy efficiency and short collection path. In this paper, we propose to design a multi-ring routing structure as shown in Figure 6.

#### 3.2.2. Build the Routing Structure

However, how to build an efficient routing structure containing rings shown in Figure 6 is a problem. Fortunately, this structure can be split into tree structures. Without loss of generality, we take the structure shown in Figure 6 as an example. For each routing path with a ring, if we cut off the ring at the target node which is the most far to sink among all target nodes on that ring, and append this target node to the two new endpoints, we can get two routing trees. Each tree is rooted at sink, and has the same target nodes as leaf nodes. These trees have different direction. One tree is used to transmit packets from the sink to each selected node, and the other is used to transmit packets toward the sink. The former is called downlink tree, and the latter is called uplink tree.

Consequently, the problem of building multi-ring routing structures is transformed into building two multicast trees. There are many classic algorithms to build multicast trees, for example [17,18]. However, it should be noted that, there is some different between the proposed multicast tree and the classic one. Firstly, each of the trees built here does not need to cover all the target nodes; only the combination of the two trees to cover all target nodes is required. This makes the optimization process more complex than in a classic one. Secondly, two trees built are node disjoint, except the leaf nodes. Finally, as the path for each packet is a ring, there is no strict upstream or downstream relationship among nodes; therefore, a new packet format must be defined.

Formal Description of the Problem (Node Disjoint Double Multicast Trees, NDDMT)

Given a graph *G* = (*V*, *E*), V = {*ν_i_*, *i* = 1,2, …, *N*} is the set of sensor nodes and *E* is the *set* of edges. Define *D* as the target nodes set. The object is to build two node disjoint multicast trees *T*_1_ *T*_2_, (*T*_1_ ∈ *V*, *T*_2_ ∈ *V*, which has minimum cost and can cover all target nodes in *D*. To build a sparse projection vector, the cost here is defined as the total number of nodes on the two multicast trees.

Define *ς_i_* to indicate whether node *v_i_* is in target nodes set *D*:

(13) $$\{\begin{array}{cc} \varsigma_{i}=1, & v_{i}\in D\\ \varsigma_{i}=0, & v_{i}\notin D \end{array}$$

Define *τ_i,j_* to indicate whether node *v_i_* is on the multicast tree *j*, *j* ∈ {1,2}:

(14) $$\{\begin{array}{cc} \tau_{i,1}=1, & v_{i}\in T_{1}\\ \tau_{i,2}=1, & v_{i}\in T_{2}\\ \tau_{i,j}=0, & \text{others} \end{array}$$

Define *η_i_* to indicate whether node *v_i_* is a leaf node:

(15) $$\{\begin{array}{ll} \eta_{i}=1, & v_{i}\;\text{is not a leaf node}\\ \eta_{i}=0, & v_{i}\;\text{is a leaf node} \end{array}$$

and, define *h_i_* to indicate the hops from *v_i_* to sink.

Then, the optimization problem can be expressed as:

(16) $$\begin{array}{c} \min|T_{1}\cup T_{2}|\\ \begin{array}{cc} \text{s}.\text{t}.\forall v_{i}\in D, & \underset{j\in \{1,2\}}{\text{∑}}\tau_{i,j}>0 \end{array}\\ \begin{array}{cc} \forall v_{i}\in T_{1}\cup T_{2}, & \eta_{i}+\underset{j\in \{1,2\}}{\text{∑}}\tau_{i,j}=2 \end{array}\\ \begin{array}{cc} \forall v_{i}\in \{v_{j}|\eta_{j}=0\}, & \varsigma_{i}\cdot (1-\eta_{i})=1 \end{array}\\ \begin{array}{cc} \forall v_{i}\in V-(T_{1}\cup T_{2}), & \underset{j\in \{1,2\}}{\text{∑}}\tau_{i,j}=0 \end{array} \end{array}$$

The object function means to find two trees with least nodes on them. Condition 1 means each node in set *D* must be covered by at least one tree. Condition 2 has two implications. For a leaf node, it must be covered by both two trees; for a non-leaf node, it is covered by only one tree. Condition 3 means that only node in the target set can be used as leaf node. Condition 4 refers to the node outside of the multicast trees.

*Property*. NDDMT problem is NP-hard.

*Proof*. Study one of its sub-problems. If this sub-problem is NP-hard, then the original problem is NP-hard. Let's reduce the optimization space to the problem of building two trees separately. First, we build the downlink tree *T*_1_ to cover all the nodes in *D*, which is denoted as problem *P*_1_. Then, we build the uplink tree *T*_2_ to cover the leaf nodes of *T*_1_, which is denoted as problem *P*_2_. Since both problem *P*_1_ and *P*_2_ are standard Steiner tree problems [19], which is NP-hard, NDDMT is NP-hard.

Algorithm to Build NDDMT

Since the NDDMT problem is NP-hard, we design a two steps heuristic algorithm to solve this problem.

Firstly, in graph *G* = (*V*, *E*), build multicast tree *T*_1_ rooted at the sink to cover all target nodes. Secondly, in graph *G′* = (*V′*, *E′*), build a tree *T*_2_ which can cover all leaf nodes on *T*_1_, where *V′* is the set of nodes in *V* except non-leaf nodes on tree *T*_1_. The optimization object for each tree is to minimize the number of nodes on it. Since the nodes on tree *T*_1_ is excluded when building tree *T*_2_, the two trees are node disjoint. As sensor nodes are always densely deployed and the target nodes set *D* is always small, so after deleting nodes on tree *T*_1_, the nodes in *V′* still have a high probability to build tree *T*_2_. If some nodes in the graph are unconnected when building tree *T*_2_, we can reuse nodes in tree *T*_1_ to ensure the success of the building process.

The Steiner tree building problem is to let nodes on the tree share their path as much as possible, so as to minimize energy cost. There are many classic Steiner tree building algorithms, such as MPH [17], ADH [18]. In this paper, if we define the cost of each edge to be 1, then the problem of building Steiner tree with minimum cost is equivalent to the problem of building a multicast tree with minimum nodes.

Uplink tree (*T*_1_) building algorithm:

Let *T*_1,_*_t_* denote the partial tree built at step *t*, and *C*_1,_*_t_* be the cost of *T*_1,_*_t_*. Let *D*_1,_*_t_* denote the set of target nodes that haven't joined tree *T*_1_ till step *t*. Denote *s* to represent sink.
*INPUT*: graph *G* = (*V*, *E*) , target nodes set *D*.
*OUTPUT*: tree *T*_1_ and its cost *C*_1_.
  (1) Initially, *t* = 0, *T*_1,0_ = {*s*}, *C*_1,0_*D*_1,0_ = *D*.
  (2) At step *t*, for each *v_i_* ∈ *D*_1,_*_t_*, use Dijkstra algorithm to calculate its cost (denoted as *c_i_*) to tree *T*_1,_*_t_* Define $T_{1,t}^{'}$ to be a virtual tree if *v_i_* joins *T*_1,_*_t_*, then the total cost of $T_{1,t}^{'}$ is *C*_1,_*_t_* + *c_i_*.
  (3) Based on their distance to $T_{1,t}^{'}$, sort the reminder target nodes in (*D*_1,_*_t_* − *v_i_*) from near to far, and calculate the total cost *c̄_i_* for them to join tree $T_{1,t}^{'}$.
  (4) Choose the node *v_i_* which can minimize *C*_1,_*_t_* + *c_i_* + *c̄_i_* to join tree *T*_1,_*_t_*.
  (5) Let *t* = *t* + 1, *D*_1,_*_t_* = *D*_1,_*_t_* − *v_i_*, go to (2) unless all target nodes have joined the tree.
  Building the algorithm for tree *T*_2_ is similar to *T*_1_. It is omitted here due to space limitations.

##### Complexity

First, calculate the shortest path from each target node to every other node in advance. The complexity for this step is *O*(*LN*)^2^. In the process of multicast tree building, each iteration will try to add one target node to the tree; computational complexity for each iteration is *O*(*L*^2^). In total there are *L* iterations, so the computational complexity for all iterations is *O*(*L*^3^). In conclusion, the computational complexity for double multicast trees building is *O*(*LN*^2^ + *L*^3^).

#### 3.2.3. Packet Structure

Assume the wireless channel is ideal. From the previous subsection, we are informed that in compressive sensing, one routing structure will be maintained only during one collection round, and there is no distinct downstream or upstream relationship between nodes on a path. Therefore, the routing problem here is different from that in a classic WSN. To solve this problem, a new data packet structure is required.

**Figure 7.**
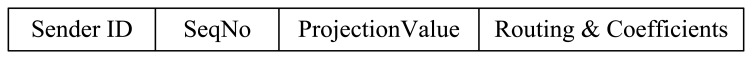
Packet structure.

**Figure 8.**
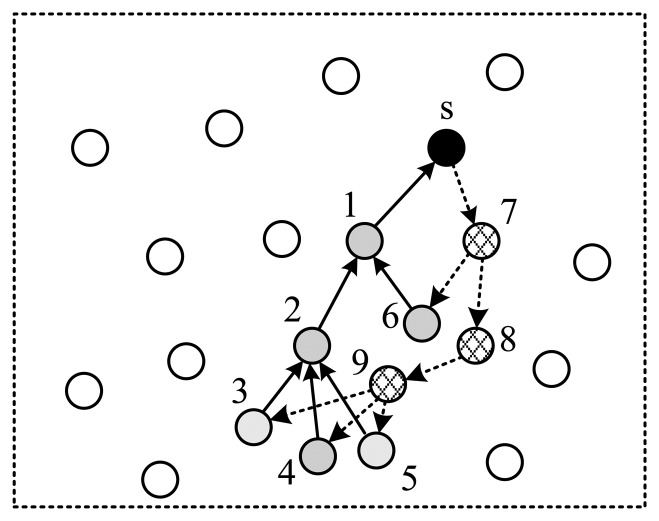
An example of the routing structure.

As is shown in Figure 7, the *SenderID* field contains the ID of the node that sends the current packet. *SeqNo* field is used to uniquely identify one collection round. Due to the broadcast nature of the wireless channel, all neighbors of a sender can receive its packet. This feature can be utilized to improve energy efficiency. When receiving a packet, a node (assumed to be *ν_i_*) checks the *Routing & Coefficients* field to determine if it is one of the children of the sender. If it is false, the packet will be dropped, otherwise *ν_i_* finds out its projection coefficient *r_i_* in the packet and updates the *Projection Value* field using:

(17) $${\text{projection}}_{\text{new}}=({\text{projection}}_{\text{old}}+x_{i}\cdot r_{i})/\text{chil}d_{i}$$

where *child_i_* indicates the number of children of *v_i_*. The reason to divide the projection value by *child_i_* is that the packet from *v_i_* will be received by all its children, which is illustrated in Figure 8 as an example.

Next, *v_i_* updates the *Routing & Coefficients* field by deleting the expired information, and broadcasts the packet to its children. (Table 1 illustrates the routing & coefficients table built from Figure 8). If a node is the child of multiple nodes, it reunites the packets received from all of its parents to one packet before sending it to its child.

#### 3.2.4. Determine Projection Coefficients

Once the nodes for one collection round are determined, we need to compute the projection coefficients for them. Equation (9) can be used to accomplish this task. Due to the constraint of sparseness, projection vector **r***_M_*__0_ + 1_ has only small part of elements with non-zero value. To obtain **r***_M_*__0_ + 1_, first delete the rows and columns in **VV***^T^***Ψ**Σ**Ψ***^T^***VV***^T^* and **VV**^T^ corresponding to the position of unselected nodes; then use the shrunk matrix pencil to determine the non-zero elements in **r***_M_*__0_ + 1_.

## 4. Simulation and Discussion

### 4.1. Simulation Scenarios

Assume nodes are uniformly distributed. The sink is located at the center of the area. The normalized communication radius of each node is *r*_c_, *r*_c_ = 1.5. Assume the communication media is ideal, with no channel noise or access collision. To evaluate the proposed algorithm, three related algorithms are selected as reference, including adaptive CS [16], adaptive Laplace BCS (adaptive BCS) [20], and Compressed Sensing with Random Walk routing (CS-RW) [21].

In the simulation, the maximum length of observation vector **y** is *M* = 120. M includes two parts: (1) initial observation vector with length *M*_0_; (2) adaptive observation vector with length *M* − *M*_0_. The initial observation vector is used as the basis for the adaptive collection stage. For adaptive BCS, the initial projection matrix is generated at the sink before collection, and then transmitted to all sensor nodes using a traditional routing mechanism. During the adaptive collection process, for each collection round, the sink determines a projection coefficient for each node and sends it out using the same routing path. For CS-RW, the probability to choose a node is fixed at 0.5.

Data used in simulation is generated using a computer. The model for data generation only assumes the data is compressible, while its sparse structure is unknown [22]. It is described as follows: assume nodes are uniformly distributed in the area, and *d_i,j_* is the distance between node *i*, *j*. Define the correlation between data from node *i*, *j* to be *c_i,j_*_=_ *_e_*^−^*^βdi,j^*, where *β* indicates the degree of correlation. Then, the matrix **C** describes the correlation is:

(18) $$C=\left[\begin{array}{cccc} c_{11} & c_{12} & \ldots & c_{1N}\\ c_{21} & c_{22} & \ldots & c_{2N}\\ \vdots & & & \vdots \\ c_{N1} & c_{N2} & \ldots & c_{NN} \end{array}\right]$$

Perform Cholesky decomposition to, **C**:

(19) $$C=G\cdot G^{T}$$

and assume **n**_0_ ∈ **R***^N^* is a uniform random vector, with each element independent and identically distributed. Then, data vector **x** is generated as:

(20) $$x=G\cdot n_{0}+n_{s}$$

where **n***_s_* denotes sensing noise.

### 4.2. Evaluation Metric

#### 4.2.1. Reconstruction Error

Assume the original data vector is **x** (*x_i_*, 1 ≤ *i* ≤ *N*), and the reconstruction data vector is **x̂** (*x̂_i_*,1 ≤ *i* ≤ *N*), then the reconstruction error is defined as:

(21) $$\text{err}=\frac{{\left\|x-\hat{x}\right\|}_{2}^{2}}{\|x{\|}_{2}^{2}}=\frac{\overset{N}{\underset{i=1}{\text{∑}}}{\left(x_{i}-{\hat{x}}_{i}\right)}^{2}}{\overset{N}{\underset{i=1}{\text{∑}}}{x_{i}}^{2}}$$

#### 4.2.2. Energy Cost

Energy cost is a key metric for wireless sensor networks. In the simulation, only the energy used in data transmission is counted, and the energy used for sensing and computation is not included. Energy consumed by the sink is not included either. Assume the transmission power is fixed, then the energy cost of a node is proportional to the number of bytes it sent.

#### 4.2.3. Computation Complexity

For compressive sensing, a node only needs to perform one multiplication and one addition operation in one projection round; therefore, the computational complexity for a node is negligible. However, the computation complexity for the sink, which includes three parts: BCS reconstruction, projection route building and projection vector computation, is much higher. In the simulation, the time elapsed in one simulation is used as metric to evaluate an algorithm's computational complexity. The simulation performed on a PC equipped with an Intel E2200 dual core processor.

### 4.3. Simulation Results

#### 4.3.1. Reconstruction Error

**Figure 9.**
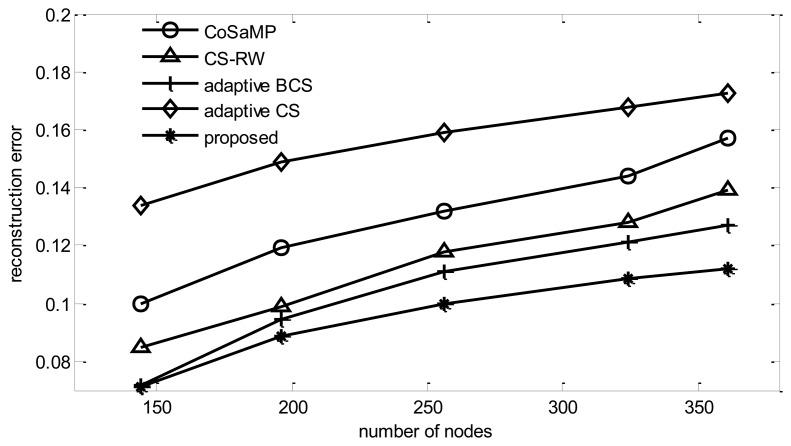
Reconstruction error *vs.* number of nodes.

From Figure 9, it can be found that, when the data is contaminated by noise, reconstruction Bayesian-based algorithms outperform CoSaMP [23]. Meanwhile, when the number of collection rounds is fixed, the reconstruction error will increase with the increase of node number *N*. Among all five algorithms, the proposed algorithm performs the best. When *N* = 400, it can reduce the reconstruction error by 2.3% in comparison with adaptive BCS. Although the adaptive method is utilized in adaptive BCS too, its performance is worse than that of the proposed algorithm, due to the fact that the Bayesian learning process is likely to choose the same basis, which leads the algorithm to fall into a local minimum [24]. Although a random noise disturbance can be added to the vector to help the learning process jump out of local minima, the performance improvement is limited while the energy cost increases dramatically. In the figure, it can be found that adaptive CS always performs the worst among the five algorithms. This is due to the fact that this algorithm tends to choose only one node in a sink's one hop neighbors as its target node. The reason for this is that adaptive CS uses Δ*h*(**p**) / Δ*E*(**p**) as a metric to select the path. As *E*(**p**) is counted in the hop, when the path length increases from 1 hop to 2 hop, *E*(**p**) increases once. However, as data from neighbor nodes has a strong correlation, in most cases, the increase of information Δ*h*(**p**) can't increase once. Therefore, adaptive CS tends to choose only one node among the sink's one hop neighbors.

#### 4.3.2. Energy Cost

**Figure 10.**
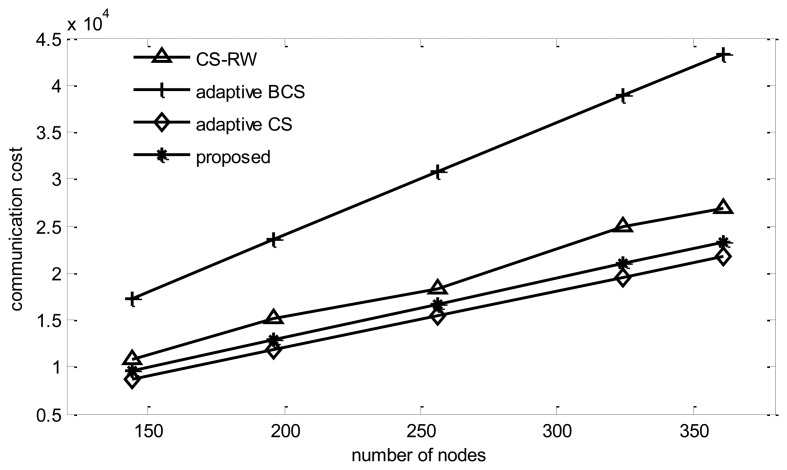
Communication cost *vs.* number of nodes.

It can be found from Figure 10 that the cost of adaptive BCS surpasses the others.

This is due to the fact adaptive BCS adds a random disturbance noise to improve the performance, which leads to a dense projection matrix. Meanwhile the projection vector built by the proposed algorithm has the majority part of elements in the projection vector as zeros, and can save 50% energy cost compared to adaptive BCS. As shown in the figure, adaptive CS consumes the minimum energy among all the algorithms, since it only collects information from one node in most collection rounds. The proposed algorithm consumes 8% more energy than adaptive CS, while the improves reconstruction performance by 6%.

#### 4.3.3. Computation Complexity

**Figure 11.**
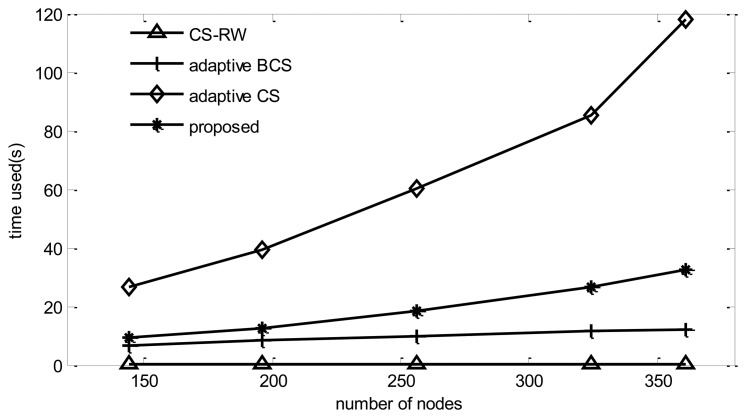
Computation complexity *vs.* number of nodes.

Since CS-RW does not use the adaptive mechanism and only needs to perform one reconstruction procedure, its simulation time is the lowest among the four algorithms. Adaptive BCS performs one reconstruction for each collection round, so its simulation time increases dramatically compared to BCS, while the adaptive CS, in addition to performing the reconstruction for each collection round, should calculate routing and projection vectors, so its simulation time increases dramatically with the number of nodes. From Figure 11, it can be found that the computational complexity of the proposed algorithm is much lower than that of adaptive CS.

#### 4.3.4. Impact of Initial Observation Length (*M*_0_)

**Figure 12.**
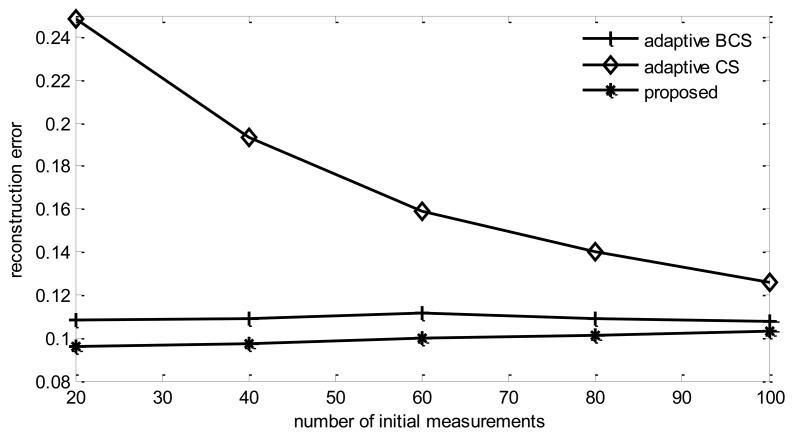
Reconstruction error *vs.* initial observation length.

From Figure 12, it can be found that the reconstruction error of all listed algorithms increases with the increase of *M*_0_.

This is due to the fact that if the total round of observations is fixed, increasing initial observations length will decrease the number of adaptive collection rounds, therefore, the performance will be degraded. It also can be found that the reconstruction error of adaptive CS will reduce with the increase of the initial observation length. This is also due to the reason explained previously. Adaptive CS tends to choose only one node in sink's one hop neighbors to collect information, therefore, the more rounds of adaptive collection performed, less information will be collected.

#### 4.3.5. Impact of Target Node Selection Metric

**Figure 13.**
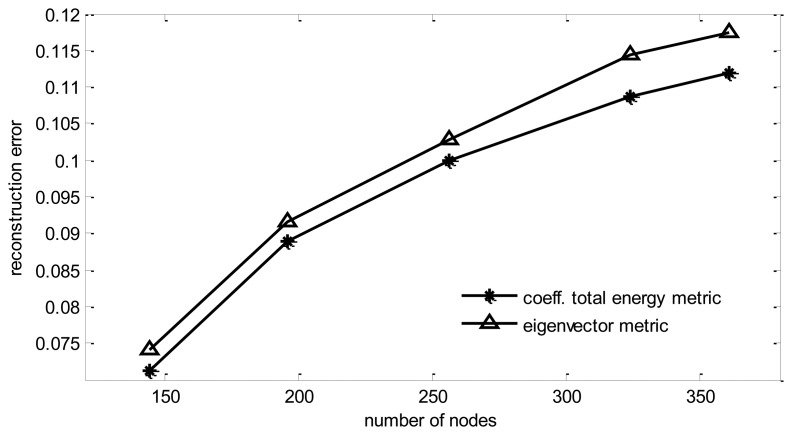
Reconstruction error for different node selection metrics.

In this paper, two target nodes selection metric are proposed. Here we compare their performance. It can be found from Figure 13 that, the reconstruction error using node's total coefficients energy as metric is smaller than using principle components of the eigenvector as metric. The reason lies in that the Bayesian learning process tends to select the same basis, which will reduce the difference between projection vectors and degrade reconstruction performance. On the contrary, node's total coefficients energy metric always can build a different projection vector.

#### 4.3.6. Number of Target Nodes

**Figure 14.**
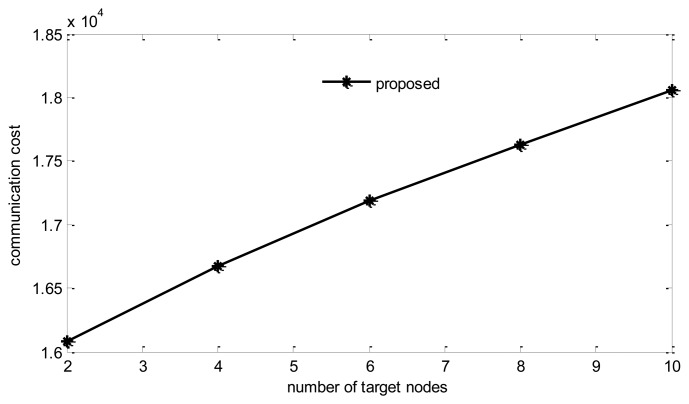
Energy cost *vs.* number of target nodes.

**Figure 15.**
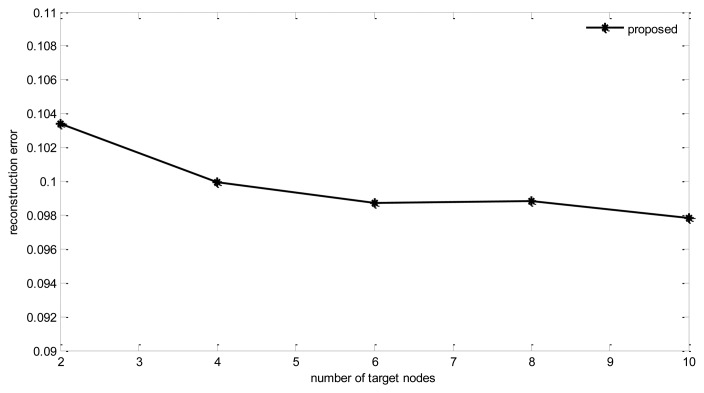
Reconstruction error *vs.* number of target nodes.

Figures 14 and 15 show the impact of the target nodes number for tree building.

It can be found that with the increase of target nodes, there is some degree of decrease in reconstruction error. However, the communication cost increases rapidly too. From the figure we can find that, when target nodes increase from 2 to 10, communication costs increase by 12%, while the reconstruction error only decreases 0.6%. Therefore, a proper number of target nodes should be selected. In the simulations, this number is fixed at four.

#### 4.3.7. Noise Power

**Figure 16.**
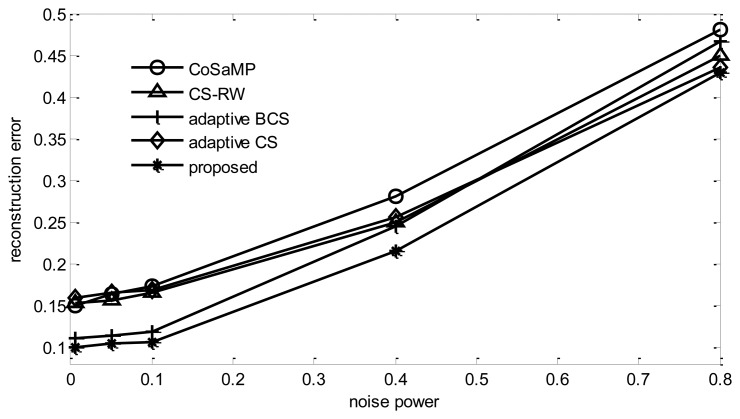
Impact of noise power.

From Figure 16, it can be found that, with increasing noise power, the reconstruction error of all algorithms will increase. Among them, the proposed algorithm has the best reconstruction performance in all experiments.

## 5. Conclusions

In this paper, a new adaptive data collection algorithm based on a Bayesian compressive sensing framework is proposed. Since the problem of finding the optimum projection vector with routing structure embedded is NP-hard, we propose a heuristic algorithm with three steps, including target nodes selection, routing structure building and projection coefficients computation. Firstly, by defining two metrics, named principle component of eigenvector and node's total coefficients power, the sub-problem of target nodes selection is solved. Secondly, we transform the problem of routing structure building to the problem of building double node disjoint broadcast trees. Finally, utilizing the theory of maximizing the differential entropy, the optimum coefficient for each selected node is obtained. Simulation results show that, compared with the reference algorithms, the proposed algorithm has better reconstruction performance with lower communication cost. Future research will study the problem of adaptive compressive sensing in a lossy communication medium and to parallelize the reconstruction process in a multi-core processor platform.

## Figures and Tables

**Table 1.** Routing & coefficients table of Figure 8.

**Node**	**Upstream**	**Downstream**	**Coeff.**
s	1	7	N.A.
1	2,6	s	r1
2	3,4,5	1	r2
3	9	2	r3
4	9	2	r4
5	9	2	r5
6	7	1	r6
7	s	6,8	r7
8	7	9	r8
9	8	3,4,5	r9
